# Self-Reported Total Screen Time and Viewing Modes Are Associated with Body Dissatisfaction, Disordered Eating, and Cosmetic Surgery Intentions among Young Adults

**DOI:** 10.3390/nu14102027

**Published:** 2022-05-12

**Authors:** Lisa Tang, Sheryl L. Rifas-Shiman, Alison E. Field, S. Bryn Austin, Jess Haines

**Affiliations:** 1 Department of Family Relations and Applied Nutrition, University of Guelph, Guelph, ON N1G 2W1, Canada; jhaines@uoguelph.ca; 2 Department of Population Medicine, Harvard Medical School, Harvard Pilgrim Health Care Institute, Boston, MA 02215, USA; sheryl_rifas@harvardpilgrim.org; 3 Department of Epidemiology, School of Public Health, Brown University, Providence, RI 02903, USA; alison_field@brown.edu; 4 Department of Social and Behavioral Sciences, Harvard T.H. Chan School of Public Health, Boston, MA 02115, USA; bryn.austin@childrens.harvard.edu; 5 Channing Division of Network Medicine, Brigham and Women’s Hospital, Harvard Medical School, Boston, MA 02115, USA; 6 Division of Adolescent/Young Adult Medicine, Boston Children’s Hospital, Boston, MA 02115, USA

**Keywords:** screen-time, body image, body dissatisfaction, cosmetic surgery intention

## Abstract

While numerous studies have shown that media exposure is linked to body dissatisfaction and disordered eating behavior, limited research has examined these associations by screen-viewing mode. This study examined associations of total screen-time and screen-viewing modes with body dissatisfaction, disordered eating, and cosmetic surgery intention among young adults. Men (*n* = 3466) and women (*n* = 7300), aged 19 to 34 years, self-reported their screen-time on various TV viewing modes, and their body dissatisfaction, overeating, disordered weight control behaviors, and cosmetic surgery intentions. We fit linear, logistic, and multivariate models to examine cross-sectional associations between total screen-time and screen-viewing modes and body dissatisfaction, disordered eating, and cosmetic surgery intention. Handheld viewing was associated with body dissatisfaction for women only, and online viewing was associated with greater body dissatisfaction among both men (βˆ = 0.40; 95% CI, 0.15 to 0.65) and women (βˆ = 0.25; 95% CI, 0.10 to 0.40). Downloaded viewing was associated with higher odds of overeating behaviors among both men (OR = 1.24; 95% CI, 1.10 to 1.40) and women (OR = 1.21; 95% CI, 1.12 to 1.32), respectively. Although total screen time was associated with greater cosmetic surgery intention for both men (βˆ = 0.24; 95% CI, 0.09 to 0.39) and women (βˆ = 0.43; 95% CI, 0.28 to 0.58), sex differences were found for the viewing modes. Our results suggest that different viewing modes may be differently associated with men and women’s body image, disordered eating behavior, and cosmetic surgery intention. Future research should consider all modes of screen-viewing in our media environment.

## 1. Introduction

Body dissatisfaction, defined as a person’s negative subjective evaluation related to the weight and shape of their own body [1], continues to be a growing public health concern [2] affecting both men and women across age, ethnicity, and weight status [3,4,5,6]. Research suggests that up to 70% of young adult women and 60% of young adult men report being dissatisfied with their bodies [5,6]. Feelings of body dissatisfaction are associated with higher rates of dieting and disordered eating behaviors, which can lead to an increased risk of developing an eating disorder [7], as well as greater weight gain among those who have overweight or obesity [8,9,10,11]. This can have an important impact on health care costs in the United States, with research showing an increased health care cost of USD $1869 per person with an eating disorder compared to those without [12]. A 10-year longitudinal study in the United States found that body dissatisfaction increased as females and males transitioned from adolescence into young adulthood [13], highlighting the need to understand key risk factors for dissatisfaction among young adults.

Research suggests young adults’ perception and satisfaction with their body shape and size are influenced by a number of psychological and sociocultural factors [14]. Images of unrealistic and idealized body types for both men and women are ubiquitous in print and screen-based media. Screen time is typically defined as time spent on activities done in front of a screen including watching television (TV), working on a computer, or playing video games [15]. Cross-sectional studies have found a positive association between media exposure and body dissatisfaction and disordered eating behaviors [16,17,18]. Research has also shown that exposure to media (television and advertising) predicts interest in cosmetic surgery [19]. Cosmetic surgery reality shows, such as “Extreme Makeover”, which aired on broadcast TV from 2002–2007, or the “The Swan”, which aired on broadcast TV in 2004, normalized cosmetic surgery as a way to correct perceived appearance imperfections. During the time that these shows aired, the American Society for Aesthetic Plastic Surgery reported substantive increases in the number of cosmetic procedures performed among young adults [20]. These shows were also available on streaming services, such as Hulu. Television makeover shows have continued to remain popular. More recent examples include shows such as “10 Years Younger in 10 Days”, a British makeover show that first aired in 2004 and relaunched in 2020 [21], and “Dr. 90210”, an American plastic surgery reality makeover show that aired from 2004 to 2008 and returned for a seventh season in 2020 featuring an all-female surgeon cast [22]. These TV shows are broadcast on basic cable networks and can also be streamed online.

The vast majority of studies investigating associations between screen time and body dissatisfaction, disordered eating, and cosmetic surgery intention focus only on exposure to traditional forms of media, i.e., television/DVD and computer [16,17,23,24], with few studies considering video viewing/streaming via mobile media devices [25]. The PEW Research Centre (2019) reported that 99% of Americans 18–29 years of age own a smartphone [26] and research has shown that young adults prefer online streaming services to watch television over a traditional cable or satellite subscription [27]. A recent Common Sense Media report identified that no research has explored the association between video viewing/streaming via mobile devices and body image [28]. Given that online advertising can be targeted using browser history and demographic information, use of online streaming may have even more detrimental impacts on body image, risk of disordered eating, and cosmetic surgery intention than traditional forms of media as advertisers may target advertisements that promote unrealistic and idealized body types to youth and young adults [28]. Further, the majority of published research that investigates the influence of screen time on body dissatisfaction, disordered eating, and cosmetic surgery intention in young adults is focused on women. However, a substantial number of men experience body dissatisfaction [29] and suffer from disordered eating behaviors [30,31]. Thus, research that considers associations between online viewing/streaming via mobile media devices on body image, disordered eating behavior, and cosmetic surgery intention among both males and females is needed. 

Using data from a large, national cohort study of young adults that measured television (TV) viewing exposure from traditional and mobile forms of screens, the current study aimed to address these gaps by examining the cross-sectional associations of screen time, assessed across different types of screen-based devices (television, mobile devices, and computer) with body dissatisfaction, disordered weight control behaviors (DWCB) (specifically use of diet pills, laxatives, and purging behavior), and cosmetic surgery intentions. This study also explored the extent to which associations of screen time with these outcomes differ across the viewing modes (broadcast, recorded, online, downloaded, and hand-held device). Results from this study provide a clearer understanding of the associations between time spent on all screen-based devices and body dissatisfaction, disordered eating behaviors, and cosmetic surgery intentions among both males and females. These findings can help guide future media literacy and eating disorder prevention interventions.

## 2. Materials and Methods

### 2.1. Study Population

Participants were from the US Growing Up Today Study (GUTS), a prospective cohort of children of women in the Nurses Health Study 2 (NHS2). The GUTS cohort was initiated in 1996 with 16,882 children (7843 males and 9039 females) aged 9–14 years (GUTS1) and expanded in 2004 with the addition of 10,923 children (4916 males and 6004 females), aged 9–16 years (GUTS2). Originally, questionnaires were sent to GUTS1 and GUTS2 in alternating years, but since 2013 a single annual survey has been sent to all GUTS1 and GUTS2 participants. For this analysis, we used data from the 2014 questionnaire, which included measures of screen viewing, body image, and cosmetic surgery intention. Questionnaires (*n* = 13,580) were received by 4702 males and 8878 females. Participants with missing data (1236 males and 1578 females) were excluded from these analyses; thus, our final analytic sample included 3466 males and 7300 females. This study was approved by the Brigham and Women’s Hospital Institutional Review Board and followed the Strengthening the Reporting of Observational Studies in Epidemiology (STROBE) reporting guidelines for cross-sectional studies.

### 2.2. Measures

#### 2.2.1. Predictor Variables

##### Screen Time and TV Viewing Modes

In this study, screen time refers to television (TV) specific screen viewing and does not include time spent playing video games. Screen time was self-reported and measured by asking participants the number of hours per week spent viewing TV shows or movies on each of the following TV viewing modes: broadcast, recorded (e.g., digital video recorder or Tivo), online (e.g., Hulu), downloaded (e.g., On Demand or Netflix), and hand-held devices (e.g., tablet or smartphone). For each viewing mode, participant response options ranged from 0 to 61+ h per week.

#### 2.2.2. Outcome Variables

##### Weight Modification

Weight modification was measured using a single question, “which of the following are you currently trying to do about your weight?” Response options included “nothing”, “stay the same”, “gain weight”, “lose weight”, and coded as 0 (nothing/stay the same), 1 (lose weight), and 2 (gain weight), with nothing/stay the same as the reference.

##### Overeating Episodes

Overeating was measured using a single question, “sometimes people will go on an ‘eating binge’ when they eat an amount of food that most people, like their friends would consider to be very large, in a short period of time. In the past year, how often did you go on an eating binge?”. This item was adapted from the Youth Risk Behavior Surveillance System questionnaire [32] and has been validated within the GUTS cohort [33]. Response options were “never”, “less than monthly”, “1–3 times per month”, “once a week”, “more than once a week”. The response “never” and “less than monthly” was coded as 0 (no overeating), and all remaining responses were coded as 1 (overeating).

##### Disordered Weight Control Behaviors (DWCB)

Disordered weight control behaviors (DWCB) was assessed with three questions. The root to the question was “in the past year, did you do any of the following to lose weight or keep from gaining weight?”, followed by the three questions “use diet pills”, “make yourself throw up”, and “take laxatives”. These questions were adapted from the Youth Risk Behavior Surveillance System questionnaire [32] and has been validated within the GUTS cohort [33]. For each of the questions, response options were “never”, “less than monthly”, “1–3 times per month”, “once a week”, and “more than once a week”. The responses “never” and “less than monthly” were coded as 0 (no DWCB), and all remaining responses were coded as 1 (DWCB in the past month). If participants scored 1 for any of the three questions (diet pills, make yourself throw up, laxatives), then they were coded as DWCB in the past month.

##### Body Dissatisfaction

Body dissatisfaction was measured using the following questions from the Overconcern with Weight and Shape Scale from the McKnight Risk Factor Survey: [34] “In the past 12 months, how often have you worried about having fat on your body?”, “In the past 12 months, how often have you thoughts about wanting to be thinner?”, and “In the past 12 months, how often have you felt fat?”. [34] The question “In the past 12 months, how often have you thought about wanting to have toned or defined muscles?” was added to improve suitability of the body dissatisfaction measure among males, who have different body ideals than females [35]. Response options were “never”, “a little”, “sometimes”, “a lot”, and “always”, and coded as 1, 2, 3, 4, and 5 respectively. Scores were then summed with the lowest possible score as 4 and the highest possible score as 16, with lower scores indicating less body dissatisfaction.

##### Cosmetic Surgery Intentions

Cosmetic Surgery Intentions, defined as intention to undergo cosmetic surgery, were measured using five questions from the Acceptance of Cosmetic Surgery Scale. [36] Questions included “I have sometimes thought about having cosmetic surgery”, “If I could have a surgical procedure done for free, I would consider trying cosmetic surgery”, “If I knew there would be no negative side effects or pain, I would like to try cosmetic surgery”, and In the future, I could end up having some kind of cosmetic surgery”, and “I would never have any kind of cosmetic surgery” (reverse coded). [36] Response options were on a 7-point Likert scale ranging from strongly disagree to strongly agree, and were coded as 1, 2, 3, 4, 5, 6, and 7 respectively. Scores were then summed with the lowest possible score as 5 and the highest possible score as 35, with higher scores meaning more cosmetic surgery intentions.

#### 2.2.3. Covariates

##### Body Mass Index and Age

Age in years was calculated using participant’s birthdate and the date, the 2014 questionnaire was collected. Body mass index (BMI) (kg/m^2^) was calculated from self-reported height and weight measures collected in the 2014 questionnaire.

#### 2.2.4. Statistical Analysis

To determine total screen time per day, participant responses were divided by 7 to calculate daily screen time spent on each viewing mode, and then summed to estimate the total daily screen time inclusive of all viewing modes. To account for correlation between siblings, linear regression using generalized estimating equations (GEE) was used to examine cross-sectional associations between total daily screen time and body dissatisfaction, and cosmetic surgery intentions. Linear regression models using GEE was also used to examine associations between each TV viewing mode (broadcast, recorded, online, downloaded, and hand-held device) and body dissatisfaction, and cosmetic surgery intentions. Logistic regression using GEE was used to examine associations between total daily screen time and overeating and DWCB and to examine associations between each viewing mode and overeating and DWCB.

Multivariate models using GEE were run to examine associations between total daily screen time and weight modification and each viewing mode and weight modification. All models were stratified by sex based on evidence that level of disordered eating differs by sex [37]. All models were adjusted for age and BMI^3^ for males and BMI^2^ for females [38]. Research from Calzo et al. (2012) found differences when comparing reported feeling of body dissatisfaction among males and females [38]. Specifically, greater body dissatisfaction was reported in females who were above the 50th for BMI compared to those below the 50th percentile [38]. However, males who were either below the 10th percentile or above the 75th percentile reported the most body dissatisfaction [38]. Thus, BMI was analyzed as quadratic and cubic polynomial functions to account for sex differences in body dissatisfaction as previous research has shown the relationship between BMI and body dissatisfaction is curvilinear [38]. BMI and age were included as covariates in all models as media can impact weight concern differently according to a person’s obesity status [18], and the association between media exposure and eating behavior differs by age [39].

## 3. Results

Participant age ranged from 19–34 years with a mean age of 28.2 years (Standard Deviation = 3.4), 95.4% were non-Hispanic white, and 67.8% were female (Table 1). The average total screen time was 1.8 (1.8) hours/day for men and 1.6 (1.5) hours/day for women and average viewing times were similar across the screen-viewing modes. More women reported trying to lose weight as compared to men (56.7% vs. 40.5%, *p* < 0.0001) (Table 1). Compared to men, more women reported having engaged at least once in the past month in DWCB (4.5% vs. 1.2%, *p* < 0.0001) and slightly more women reported engaging in overeating in the past month (10% vs. 9.1%, *p* = 0.13) (Table 1). Correlations between the outcomes ranged from 0.001–0.35 with the strongest correlation found between body image and cosmetic surgery intention (data not shown).

### 3.1. Body Dissatisfaction

For women, total screen time (βˆ = 0.18; 95% CI, 0.12 to 0.24), downloaded (βˆ = 0.23; 95% CI, 0.10 to 0.35), and handheld device (βˆ = 0.42; 95% CI, 0.16 to 0.68) were positively associated with body dissatisfaction. Online viewing was positively associated with body dissatisfaction among both women (βˆ = 0.25; 95% CI, 0.10 to 0.40) and men (βˆ = 0.40; 95% CI, 0.15 to 0.65). Recorded viewing was also positively associated with body dissatisfaction among both women (βˆ = 0.28; 95% CI, 0.14 to 0.42) and men (βˆ = 0.28; 95% CI, 0.05 to 0.51). Broadcast viewing was not found to be associated with body dissatisfaction for women or men (Table 2).

### 3.2. Weight Modification

Using stay the same/do nothing as the referent group, total screen time was associated with greater odds of trying to lose weight for both women (OR = 1.09; 95% CI, 1.04 to 1.15) and men (OR = 1.06; 95% CI, 1.01 to 1.13) and gain weight (OR = 1.17; 95% CI, 1.07 to 1.27) among women only. For women, recorded (OR = 1.14; 95% CI, 1.03 to 1.25) and downloaded (OR = 1.11; 95% CI, 1.02 to 1.21) viewing modes were associated with higher odds of trying to lose weight, and broadcast (OR = 1.31; 95% CI, 1.07 to 1.61) and recorded (OR = 1.52; 95% CI, 1.23 to 1.87) viewing were associated with higher odds of trying to gain weight. Online (OR = 1.20; 95% CI, 1.03 to 1.39) and handheld viewing (OR = 1.54; 95% CI, 1.14 to 2.10) were associated with higher odds of trying to lose weight for men, but not women (Table 3).

### 3.3. Overeating Episodes

For women only, total screen time (OR = 1.12; 95% CI, 1.06 to 1.17) and online viewing (OR = 1.28; 95% CI, 1.15 to 1.43) were associated with higher odds of overeating. For men only, recorded viewing was associated with higher odds of overeating (OR = 1.17; 95% CI, 1.01 to 1.35). Downloaded viewing was associated with higher odds of overeating for both women (OR = 1.21; 95% CI, 1.12 to 1.32) and men (OR = 1.24; 95% CI, 1.10 to 1.40). No associations were found between broadcast and handheld viewing and overeating among women or men (Table 3).

### 3.4. Disordered Weight Control Behaviors (DWCB)

For women only, total screen time (OR = 1.10; 95% CI, 1.04 to 1.15), and the following viewing modes were associated with higher odds of DWCB: recorded (OR = 1.22; 95% CI, 1.09 to 1.36), online (OR = 1.17; 95% CI, 1.02 to 1.34), and downloaded (OR = 1.14; 95% CI, 1.01 to 1.29). No associations were found between any viewing mode and DWCB among men (Table 3).

### 3.5. Cosmetic Surgery Intention

Total screen time was positively associated with cosmetic surgery intention among both women (βˆ = 0.43; 95% CI, 0.28 to 0.58) and men (βˆ = 0.24; 95% CI, 0.09 to 0.39). However, sex differences were found when examining the individual TV viewing modes. Specifically, broadcast (βˆ = 0.54; 95% CI, 0.22 to 0.86) and recorded (βˆ = 1.32; 95% CI, 0.97 to 1.67) viewing were positively associated with cosmetic surgery intention among participant women, but not men. Conversely, online (βˆ = 0.57; 95% CI, 0.17 to 0.97), downloaded (βˆ = 0.44; 95% CI, 0.10 to 0.78), and handheld viewing (βˆ = 0.95; 95% CI, 0.23 to 1.67) were positively associated with cosmetic surgery intentions for men, but not women (Table 2).

## 4. Discussion

This study examined the associations between total screen time and the different screen viewing modes (broadcast, recorded, online, downloaded, and handheld) and body dissatisfaction, disordered eating, and cosmetic surgery intention among a national sample of young adults 19–34 years of age. Overall, this study found that screen time and screen viewing modes were cross-sectionally associated with measures of body dissatisfaction, disordered eating behavior, and cosmetic surgery intention. However, associations between viewing modes and the various outcomes differed between women and men.

Our finding that recorded and online viewing, but not broadcast, were associated with body dissatisfaction among both men and women may be explained by a difference in the content, i.e., type of shows, or in the exposure to advertising across viewing modes. For example, it is possible that watching online TV, which may include sports streaming, could potentially expose men to more images of the muscular ideal, which may lead to increased feelings of body dissatisfaction. Moreover, during the time of data collection, the online viewing platform Hulu had targeted online advertisements based on user information and survey questions such as “is this ad relevant to you?” [40], as opposed to the traditional content-based television advertising. This could have resulted in some participants identifying more body-focused advertisements, e.g., fashion or diet-related products, as relevant to them, leading to an increase in their exposure to such advertisements, which, in turn, could have influenced the impact of that viewing mode on their level of body dissatisfaction.

Total screen time was also associated with greater odds of trying to lose weight for both men and women, and with greater odds of trying to gain weight among women only. While the prevalence of trying to gain weight was low among women, with only 1.4% of female participants reporting trying to gain weight, total screen time, broadcast and downloaded viewing were all significantly associated with trying to gain weight among women. The specific reasons for this desire to gain weight among women when viewing broadcast and downloaded TV were not examined in this study; however, the content viewed on broadcast and downloaded TV may play a role. For example, research has shown that viewing sports [41,42] and news programming [41] are associated with higher body satisfaction among women. Thus, future research should examine the specific content viewed to help elucidate the relationships between screen viewing modes and body dissatisfaction.

Regarding overeating, we found similar associations between screen viewing and overeating among men and women. This is consistent with previous research that has found an association between television use and binge eating behaviors among a non-stratified adult population of men and women [43]. Sex differences were found for DWCB. Specifically, total screen time, online, downloaded, and recorded TV were found to be positively associated with DWCB for women, whereas none of these screen viewing modes were significantly associated with DWCB among men. The odds ratios for recorded viewing and DWCB were similar for men (1.21) and women (1.17); however, the smaller number of men reporting DWCB behaviors led to wider confidence intervals and results that were not statistically significant. Taken together, these results suggest that total screen time and modes of viewing may be associated with disordered weight control behaviors among both men and women; however, further research with larger samples of males is needed.

Total screen time was positively associated with cosmetic surgery intention for both men and women. However, when viewing was separated by individual TV viewing mode, sex differences were found. Specifically, broadcast (βˆ = 0.54; 95% CI, 0.22 to 0.86) and recorded (βˆ = 1.32; 95% CI, 0.97 to 1.67) viewing was positively associated with cosmetic surgery intention among participant women but not men. Conversely, screen viewing online (βˆ = 0.57; 95% CI, 0.17 to 0.97), downloaded (βˆ = 0.44; 95% CI, 0.10 to 0.78), and on a handheld device (βˆ = 0.95; 95% CI, 0.23 to 1.67) was positively associated with cosmetic surgery intentions for participant men, but not women. Our findings among women were consistent with previous research by Slevec and Tiggemann (2010) who found that TV exposure predicted the consideration of cosmetic surgery among adult women. This study also found that TV exposure was associated with women being socially motivated for cosmetic surgery [44]. Research that examines women’s viewing of cosmetic surgery related programs (shows such as *Embarrassing Bodies*) is associated with increased interest in undergoing cosmetic surgery [19,45]. Our study findings for men are consistent with a previous research study that demonstrated a link between TV viewing and men’s interest in cosmetic surgery, although this study showed that women were more interested in cosmetic surgery than men [46]. Similarly, a study by Swami et al. (2008) found that women reported a higher willingness to engage in cosmetic surgery than men [47]. Taken together, there seems to be a link between screen time exposure and cosmetic surgery intention among men and women. However, research exploring this association is limited, and research that involves men is even less present in the literature. Given our study results, further research into the role that different viewing modes may play in increasing cosmetic surgery intention among both men and women is warranted.

### 4.1. Strengths

A strength of our study includes the inclusion of both men and women in the analysis. Published research related to body image and disordered eating is limited in its representation of men. This study furthers this field of research by including men separately in the analysis. Another strength of our study is that our measures moved beyond examining only the total screen time to include individual viewing modes. This allowed us to investigate the association between media and men and women’s body dissatisfaction, disordered eating, and cosmetic surgery intention in a way that better represents our diverse media environment. Finally, research related to screen time and its influence on cosmetic surgery intention is lacking. Our study builds on previous research examining the influences on intention to undergo cosmetic surgery to examine the linkage between individual viewing modes and its influence on men and women’s cosmetic surgery intention.

### 4.2. Limitations

This study had some limitations that should be considered when interpreting our results. First, although this study population resides in the USA, study participants are sons and daughters of nurses and predominately white (95%), which limits the generalizability of study findings. Second, all measures were self-reported, which may result in social-desirability bias or errors in estimating the amount of time spent viewing each of the screen viewing modes. Although research has demonstrated that self-reported measures of screen time can be used to categorize individuals as being either high, medium, or low screen users, objective measures are needed to increase measurement precision [48]. Thus, future research should focus on utilizing device applications to gain objective measures of total screen time and time spent on different viewing modes. Third, as this study is cross-sectional, the direction of the associations is uncertain. Fourth, this study did not collect data on the specific content viewed by participants, which would be helpful in providing a fuller understanding of the potential mechanisms of influence of media on our outcome measures. Finally, data for this research were collected in 2014, and since then, there have been advances in technology (e.g., algorithms have become more precise in online streaming tools) and the use of technology has changed among the US population, including increased accessibility to mobile media devices such as smartphones [26]. Online streaming tools have increased in popularity and individual screen time may be higher now than in 2014 as a consequence of the COVID-19 pandemic. However, the viewing modes measured in this study remain popular and relevant in 2022.

## 5. Conclusions

Overall, our results suggest that total screen time and various TV viewing modes are associated with body dissatisfaction, disordered eating behavior, and cosmetic surgery intention among young adult men and women. Given that TV media has been shown to have an influence on body dissatisfaction and disordered eating behaviors among males and females [43,49,50], research that considers all modes of viewing in our diverse media environment is needed. Our novel study provides valuable information on the cross-sectional associations of screen time, assessed across different types of screen-based devices (television, mobile devices, and computer) with body dissatisfaction, disordered eating behaviors, and cosmetic surgery intentions. This study provides a clearer understanding of media’s influence as it examined the extent to which associations of screen time with these outcomes differed across the viewing modes (broadcast, recorded, online, downloaded, and hand-held device). Future research should examine longitudinal associations between screen viewing modes and body dissatisfaction, disordered eating, and cosmetic surgery intention to help clarify the direction of associations. Further, future research should also consider the specific content that participants are viewing to help provide a fuller understanding of the kinds of content that may influence body dissatisfaction, disordered eating, and cosmetic surgery intention among young adults.

## Figures and Tables

**Table 1:** Participant characteristics according to sex (*n* = 10,766).

Variable	Male	Female
	*n* = 3466	*n* = 7300
Hispanic, *n* (%)		
No	3339 (97.7)	7044 (97.6)
Yes	79 (2.3)	175 (2.4)
Race, *n* (%)		
White	3269 (95.0)	6935 (95.6)
Black	11 (0.3)	26 (0.4)
Asian	28 (0.8)	54 (0.7)
Other	133 (3.9)	241 (3.3)
Age, years (SD)	28.2 (3.5)	28.3 (3.4)
BMI, (SD)	26.2 (4.7)	25.2 (5.6)
Exposures, hours/day (SD)		
Total television viewing time	1.8 (1.8)	1.6 (1.5)
Watching TV shows or movies when they are broadcast	0.4 (0.7)	0.4 (0.7)
Watching TV shows or movies that have been recorded (e.g., DVR, Tivo)	0.4 (0.7)	0.4 (0.7)
Watching TV shows or movies online (e.g., Hulu)	0.3 (0.6)	0.3 (0.6)
Watching DVDs or downloaded TV shows or movies (e.g., On Demand, iTunes, Netflix)”	0.5 (0.7)	0.5 (0.7)
Watching TV shows, movies, videos on hand-held device (e.g., iPad) or smartphone	0.2 (0.4)	0.1 (0.3)
Outcomes		
Weight modification, *n* (%)		
Lose weight	1403 (40.5)	4128 (56.7)
Gain weight	345 (10.0)	103 (1.4)
Stay same/do nothing	1713 (49.5)	3052 (41.9)
Overeating, *n* (%)		
No	3130 (90.9)	6544 (90.0)
Yes	314 (9.1)	730 (10.0)
DWCB *, *n* (%)		
No	3358 (98.8)	6835 (95.5)
Yes	41 (1.2)	325 (4.5)
Body Dissatisfaction, mean (SD)	10.0 (3.7)	12.7 (3.8)
Cosmetic Surgery Intention, mean (SD)	10.2 (6.8)	16.1 (9.3)

* DWCB refers to Disordered Weight Control Behaviors. * Numbers do not add up to the total because of missing values.

**Table 2:** Sex-stratified associations * of television viewing time and different screen viewing modes with body. Dissatisfaction and cosmetic surgery intention among GUTS participants.

	Body Dissatisfaction		Cosmetic Surgery Intentions	
	β (95% CI)		β (95% CI)	
	Male	Female	Male	Female
Total Screen Time	0.14 (0.00, 0.27)	0.18 (0.12, 0.24)	0.24 (0.09, 0.39)	0.43 (0.28, 0.58)
Broadcast	−0.07 (−0.25, 0.11)	0.11 (−0.02, 0.24)	−0.04 (−0.33, 0.24)	0.54 (0.22, 0.86)
Recorded	0.28 (0.05, 0.51)	0.28 (0.14, 0.42)	0.30 (−0.05, 0.65)	1.32 (0.97, 1.67)
Online	0.40 (0.15, 0.65)	0.25 (0.10, 0.40)	0.57 (0.17, 0.97)	0.06 (−0.33, 0.45)
Downloaded	0.18 (0.00, 0.36)	0.23 (0.10, 0.35)	0.44 (0.10, 0.78)	0.18 (−0.11, 0.48)
Handheld	0.47 (−0.01, 0.95)	0.42 (0.16, 0.68)	0.95 (0.23, 1.67)	0.39 (−0.40, 1.17)

* We used linear regression models corrected for clustering by siblings (GEE) and adjusted all models for age and BMI^3^ for males and BMI^2^ for females.

**Table 3:** Sex-stratified associations * of television viewing time and different screen viewing modes with overeating, disordered weight control behaviors (DWCB), and weight modification.

					Weight Modification (v. Stay Same/Do Nothing)			
	Overeating		DWCB		Lose Weight		Gain Weight	
	OR (95% CI)		OR (95% CI)		OR (95% CI)		OR (95% CI)	
	Male	Female	Male	Female	Male	Female	Male	Female
Total Screen Time	1.07 (1.00, 1.16)	1.12 (1.06, 1.17)	1.06 (0.98, 1.15)	1.10 (1.04, 1.15)	1.06 (1.01, 1.13)	1.09 (1.04, 1.15)	1.06 (0.97, 1.15)	1.17 (1.07, 1.27)
Broadcast	1.00 (0.85, 1.16)	1.08 (0.97, 1.20)	0.97 (0.60, 1.58)	1.11 (0.97, 1.28)	0.87 (0.76, 1.00)	1.07 (0.99, 1.16)	1.06 (0.89, 1.26)	1.31 (1.07, 1.61)
Recorded	1.17 (1.01, 1.35)	1.08 (0.97, 1.20)	1.27 (0.93, 1.72)	1.22 (1.09, 1.36)	1.14 (0.99, 1.30)	1.14 (1.03, 1.25)	1.09 (0.90, 1.32)	1.52 (1.23, 1.87)
Online	1.10 (0.95, 1.29)	1.28 (1.15, 1.43)	1.11 (0.73, 1.67)	1.17 (1.02, 1.34)	1.20 (1.03, 1.39)	1.07 (0.97, 1.18)	0.99 (0.80, 1.21)	0.71 (0.40, 1.28)
Downloaded	1.24 (1.10, 1.40)	1.21 (1.12, 1.32)	1.22 (0.93, 1.61)	1.14 (1.01, 1.29)	1.11 (0.98, 1.27)	1.11 (1.02, 1.21)	1.08 (0.92, 1.27)	1.10 (0.80, 1.51)
Handheld	1.12 (0.91, 1.39)	1.14 (0.93, 1.38)	1.03 (0.74, 1.44)	0.98 (0.75, 1.29)	1.54 (1.14, 2.10)	1.18 (0.94, 1.50)	1.30 (0.92, 1.83)	0.20 (0.02, 1.91)

* We used logistic regression models corrected for clustering by siblings (GEE) and adjusted male models for age and BMI3 and female models for age and BMI2.

## Data Availability

GUTS welcomes outside collaborators and has an NIH approved data enclave approach for data sharing. Interested investigators can contact GUTS investigators to explore this option, which preserves participant confidentiality and meets the requirements of our Institutional Review Board, to protect human subjects. Due to Institutional Review Board restrictions and participant confidentiality, we do not make participant data publicly available.
